# Multi-omics insight into the molecular networks of mental disorder related genetic pathways in the pathogenesis of inflammatory bowel disease

**DOI:** 10.1038/s41398-025-03299-2

**Published:** 2025-03-21

**Authors:** Meng Zhang, Jianhui Zhao, Haosen Ji, Yuqian Tan, Siyun Zhou, Jing Sun, Yuan Ding, Xue Li

**Affiliations:** 1 Center of Clinical Big Data and Analytics of The Second Affiliated Hospital, School of Public Health, Zhejiang University School of Medicine, Hangzhou, 310058 China; 2 National Institute for Data Science in Health and Medicine, Zhejiang University, Hangzhou, 310058 Zhejiang China; 3 Zhejiang Key Laboratory of Intelligent Preventive Medicine, Hangzhou, 310058 Zhejiang China; 4 Department of Hepatobiliary and Pancreatic Surgery, The Second Affiliated Hospital, Zhejiang University School of Medicine, Hangzhou, Zhejiang China; 5 Key Laboratory of Precision Diagnosis and Treatment for Hepatobiliary and Pancreatic Tumor of Zhejiang Province, Hangzhou, Zhejiang 310009 China; 6 Center for Medical Research and Innovation in Digestive System Tumors, Ministry of Education, Hangzhou, China

**Keywords:** Clinical genetics, Predictive markers

## Abstract

Mental disorders are associated with inflammatory bowel disease (IBD), but the genetic pathophysiology is not fully understood. We obtained data on mental disorder-related gene methylation, expression, protein levels, and summary statistics of IBD, and performed Summary data-based Mendelian randomization and colocalization analyses to explore the causal associations and shared causal genetic variants between multiple molecular traits and IBD. Integrating multi-omics data, we found *QDPR*, *DBI and MAX* are associated with ulcerative colitis (UC) risk, while *HP* is linked to IBD risk. Inverse associations between gene methylation (cg0880851 and cg26689483) and expression are observed in *QDPR*, consistent with their detrimental role in UC. Methylation of *DBI* (cg11066750) protects against UC by enhancing expression. Higher levels of *DBI* (OR = 0.79, 95%CI = 0.69–0.90) and *MAX* (OR = 0.74, 95%CI = 0.62–0.90) encoded proteins are inversely associated with UC risk, while higher *QDPR* (OR = 1.17, 95%CI = 1.07–1.28) and *HP* (OR = 1.09, 95%CI = 1.04–1.14) levels increase UC and IBD risk. Our findings advance the understanding of IBD’s pathogenic mechanisms and gut-brain interaction.

## Introduction

Inflammatory bowel disease (IBD) represents a group of chronic systemic inflammatory conditions primarily affecting gastrointestinal tract including two clinical subtypes, Crohn’s disease and ulcerative colitis [1]. As a modern chronic disease, IBD has shown a steady increase in prevalence in the Western world since the 20th century and poses a significant public health challenge worldwide [2]. Mental disorders, e.g., major depressive disorder, anxiety, autism spectrum disorder and schizophrenia, are generally characterized by a combination of abnormal thoughts, perceptions, emotions, behavior, and relationships with others [3]. A Global Burden of Disease study shows that there are 970.1 million cases of mental disorders in 2019, with depressive and anxiety disorders remaining among the dominant causes of burden worldwide [4].

Currently, there is widespread recognition of the comorbidities and connections between gastrointestinal diseases and mental disorders [5–7]. In this context, associations between various mental disorders, such as obsessive-compulsive disorder, depression, bipolar disorder, and the onset of IBD have been established through epidemiological studies and Mendelian randomization [8–14]. Genome-wide association studies (GWAS) have identified specific genes, such as *NR5A2* and *PPP3CA*, as shared susceptibility genes with transcriptional significance for both mental disorders and IBD [15]. However, research at a specific molecular level in this context is still limited. Unraveling the genetic pathways associated with mental disorders and their downstream impact on the occurrence of IBD remains important to understand the comorbidity between mental disorders and IBD. Therefore, we aimed to explore the associations between genes/molecules related to mental disorders and the risk of IBD based on expression quantitative trait loci (eQTL), methylation quantitative trait loci (mQTL), and protein quantitative trait loci (pQTL).

Mendelian randomization (MR) analysis employs genetic variants as instrumental variables to strengthen the inference of causality between exposure and outcome. In comparison to observational studies, this method is less susceptible to confounding and reverse causation bias, as genetic variations are randomly distributed at conception and cannot be modified by the disease onset [16]. The increasing availability of large-scale GWAS and molecular QTL data enables the exploration of the causal associations between the regulation of mental disorders related genes and IBD. Summary data-based Mendelian randomization (SMR) was developed as a powerful tool to investigate the causal relationships between molecular traits and diseases, and has been widely applied in research aimed at identifying drug targets for various diseases [17, 18]. Herein, we utilized SMR to investigate the potential associations of mental disorder-related gene methylation, expression, and protein abundance with the risk of IBD. To strengthen causal inference and identify key genes, we also incorporated colocalization analyses into our study.

## Method

**Fig. 1:**
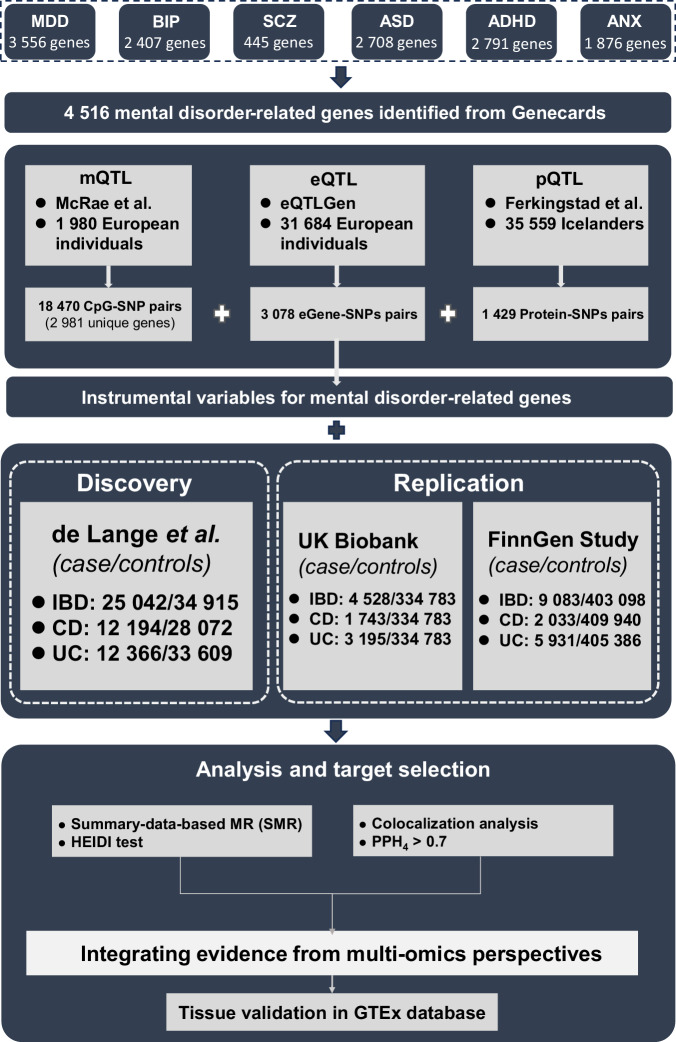
Study design flow chart. SMR summary-based Mendelian randomization, QTL quantitative trait loci, IBD inflammatory bowel disease, CD Crohn’s disease, UC ulcerative colitis, SNP single nucleotide polymorphisms, PPH4 posterior probability of H4.

Figure 1 showed the overall design of this study. All data used in our analyses were derived from former studies approved by the ethnical committees and every participant included in the studies signed an informed consent.

### Acquisition and selection of candidate genes

GeneCards (http://www.genecards.org) is a gene-centric database integrating data for more than 20 000 disease genes from multiple sources [19]. To identify mental disorder related genes, we searched on GeneCards using the terms “major depressive disorder”, “bipolar disorder”, “schizophrenia”, “autism spectrum disorder”, “attention deficit hyperactivity disorder”, “anxiety disorder” with a high relevance score ≥7 (extracted on December 31, 2023). In order to comprehensively investigate key genes involved in the gut-brain axis, we further compiled a union of genes associated with the six mental disorders. These genes were then included in the analysis as candidate genes.

### Data sources of quantitative trait loci and IBD

mQTL, eQTL and pQTL were used as instrumental variables of included candidate genes in our study. We obtained blood mQTL summary statistics from two cohorts: the Brisbane Systems Genetics Study (n = 614) and the Lothian Birth Cohorts (n = 1 366) in peripheral blood, and only SNPs (single nucleotide polymorphisms) with *P* < 1 × 10^−5^ were included [20, 21]. The methylation of all the samples were evaluated based on Illumina HumanMethylation450 chips (GPL16304) with 485 512 DNAm probes documented and Price et al. [22]. annotated the close genes for each DNA methylation probes. The blood cis-eQTL summary data were extracted from the eQTLGen consortium including 31 684 participants from 37 datasets [23]. Summary data of blood pQTL was acquired from a large plasma proteome study in 35 559 Icelanders [24].

The Genotype-Tissue Expression (GTEx) project (https://gtexportal.org/) provides genetic regulatory variants affecting gene expression across 49 tissues [25]. In the tissue validation step, we obtained tissue cis-eQTL from the GTEx database (v8 release). For validation analyses, we utilized the eQTL data in the sigmoid colon, transverse colon, and small intestine (terminal ileum).

Summary statistics for IBD and its subtypes were obtained from a large-scale GWAS study performed by de Lange et al. [26] in 2017, which contained a total sample size of 59 957 individuals in European ancestry [cases/controls for IBD: 25 042/34 915; UC: 12 366/33 609; CD: 12 194/28 072]. The summary-level data for the replication were based on the R10 data release of the FinnGen study [27] [cases/controls for IBD: 9 083/403 098; UC: 5 931/405 386; CD: 2 033/409 904] and the GWAS of UK Biobank study conducted by Lee Lab [28] [cases/controls for IBD: 4 528/334 783; UC: 3 195/334 783; CD: 1 743/334 783]. All summary statistics used in this study were derived from large-scale population-based databases, such as FinnGen and UK Biobank. Larger sample sizes help to increase statistical power, which is crucial for detecting subtle associations. All participants were of European ancestry and there was no sample overlap across the datasets.

### Summary-data-based MR analysis

Based on the theories for MR, SMR has been established to estimate and test for pleiotropic association between the molecular traits and outcome and infer putatively causal genes [29]. Compared to traditional MR method, the power of SMR can be elevated by orders of magnitude when exposure and outcome data are available from two independent samples with very large sample sizes [29]. In this study, we employed SMR to investigate the associations of mental disorder-related gene methylation, expression and protein abundance with the risk of IBD, UC and CD. The top associated cis-QTL were selected by considering a window centered around the corresponding gene (±1 000 kb) and passing a p-value threshold of 5.0 × 10^−8^. For each probe, this analysis included only SNPs with eQTL p-values <1.57 × 10^−3^, excluded SNPs with LD r^2^ between top-SNP >0.9 or <0.05, and further excluded one of each pair of the remaining SNPs with LD r^2^ > 0.9. After checking the consistency of allele frequency of each SNP between pairwise data sets (including the GWAS summary data, the eQTL summary data and the LD reference data), the SNPs with allele frequency differences >0.2 between any pair of the datasets were excluded from the analysis. Heterogeneity in dependent instruments (HEIDI) test is a method to distinguish pleiotropy from linkage [29], where *P*-value < 0.01 was considered indicative of heterogeneity and discarded from our analysis. Significance *P* value threshold of 5 × 10^−8^ was defined to select the top associated QTLs as instrumental variants in the SMR analysis. All SMR analyses were performed by SMR software version 1.3.1. Using the Benjamini-Hochberg method, we calculated the false discovery rate (FDR) with 0.05 treated as the significant threshold. Odds ratio (OR) and its 95% confidence interval (CI) were calculated by effect value and standard error generated from the analysis.

### Colocalization analysis

The colocalization of pQTL-GWAS and eQTL-GWAS was assessed, which evaluates several posterior probabilities (PP) including the following five hypotheses: 1) H0: neither trait has a genetic association in the region; 2) H1: only trait 1 has a genetic association in the region; 3) H2: only trait 2 has a genetic association in the region; 4) H3: both traits are associated, but with different causal variants; 5) H4: both traits are associated and share a single causal variant. This Bayesian framework is used to determine if an overlap of QTLs and GWAS association is consistent with a shared causal variant [30]. We extracted cis-eQTL and cis-pQTL for genes located within 1 Mb upstream or downstream of the top variant [31, 32]. Before conducting the pQTL-GWAS colocalization, we mapped the SNPs of IBD GWAS to human genome Build 38 (GRCh38) to ensure consistency with the pQTL reference genome. The eQTL-GWAS and pQTL-GWAS colocalization analyses were performed using R package ‘coloc’ with PPH4 > 0.7 as the significant cut-off, as this corresponds to a false discovery rate of <5% [33].

### Integrating multi-omics level of evidence

Upon completing the analyses mentioned above, we integrated evidence from multi-omics levels. The evidence was categorized into three tiers based on the *P*-values from the SMR analysis and PPH4 from the colocalization analysis: 1) tier 1 genes were defined as those with a gene-disease association at protein abundance levels with an FDR-corrected *P*-value < 0.05 and PPH4 for colocalization greater than 0.7, and at both methylation and expression levels with an FDR-corrected *P*-value < 0.05; 2) tier 2 genes were defined as those with a gene-disease association at protein abundance levels with an FDR-corrected *P*-value < 0.05 and PPH4 for colocalization greater than 0.7, and at methylation or expression levels with an FDR-corrected *P*-value < 0.05; 3) tier 3 genes were defined as those with a gene-disease association at protein abundance levels with an FDR-corrected *P*-value < 0.05 and PPH4 for colocalization greater than 0.5, and at both methylation and expression levels with an original *P*-value < 0.05.

### Replication and tissue validation

Replication of observed associations was conducted in participants from UK Biobank and FinnGen study to improve the credibility of the discoveries. For these tier genes unveiled in the integrating step, we employed a validation using cis-eQTL from the GTEx database across three gut tissues: transverse colon, sigmoid colon and small intestinal terminal ileum. Moreover, we conducted a meta-analysis of cis-eQTL in correlated samples using SMR software to generate intestinal meta eQTL data. In tissue validation phase, nominal *P*-value < 0.05 in SMR analysis was considered as a significant level.

## Result

Taking the union of gene sets of six mental disorders, 4 516 mental disorder-related genes were included as candidate genes (Figure S1).

### Mental disorder-related gene methylation and IBD

In total, for IBD, UC and CD, 2 127, 1 707, 2 034 CpG sites near 750, 680, 745 unique genes, respectively, passed the *P*-HEIDI threshold and nominal significance (*P* < 0.05). After multiple testing corrections, there were 509, 423, 487 CpG sites near 201, 169, 188 genes significantly associated with IBD, UC and CD risk respectively (Table S1). There were inconsistencies in the effect estimates across different CpG sites within the same gene. For instance, a one standard deviation increase in genetically predicted *BCL6* methylation at cg01598596 was linked to a reduced risk of IBD (OR = 0.83, 95% CI: 0.76–0.91), whereas at cg19774694, it was associated with an increased risk of IBD (OR = 1.15, 95% CI: 1.05–1.25). Associations for 22 CpG sites near *IRF5*, *RTEL1*, *ITGAL*, *SLC22A5*, *SMAD3*, *NOD2*, *CORO1A*, *HLA-H* were replicated in both UK Biobank and FinnGen study (Table S2).

### Mental disorder-related gene expression and IBD

**Fig. 2:**
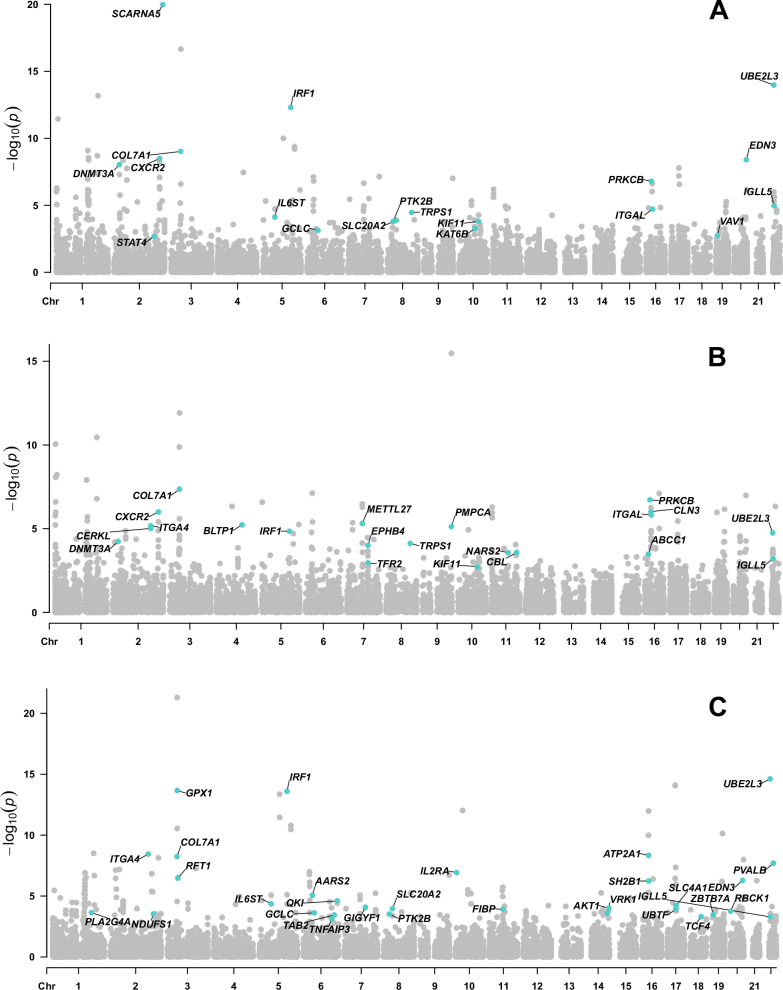
Manhattan plot for associations between genetically predicted mental disorder-related gene expression and risk of IBD A, UC B and CD C. Genes passing multiple corrections (*P* < 0.05) and colocalization threshold (PPH4 > 0.7) were labled. IBD inflammatory bowel disease, CD Crohn’s disease, UC ulcerative colitis.

After multiple testing corrections for *P*-value, 101, 65, 83 associations were identified between gene expression and risk of IBD, UC and CD separately. Moreover, 18, 21, 31 gene expressions had the evidence of multiple correction in SMR analyses and colocalization for IBD, UC and CD respectively (Fig. 2 and Table S3). For example, *PRKCB* expression was positively associated with the risk of IBD (OR = 1.13, 95%CI: 1.08–1.18, PPH4 = 0.93) and UC (OR = 1.17, 95%CI: 1.10–1.23, PPH4 = 0.93). Genetically predicted *AKT1* expression was associated with an increased risk of CD (OR = 1.26, 95%CI: 1.12–1.42, PPH4 = 0.92). In contrast, *DNMT3A* showed a protective effect against IBD (OR = 0.47, 95%CI: 0.37–0.61, PPH4 = 0.93) and UC (OR = 0.53, 95%CI: 0.39–0.72, PPH4 = 0.93). In the replication stage, associations for *ITGAL*, *IRF1*, *BLTP1* and *CORO1A* were replicated in both datasets (Table S4).

### Mental disorder-related protein and IBD

**Fig. 3:**
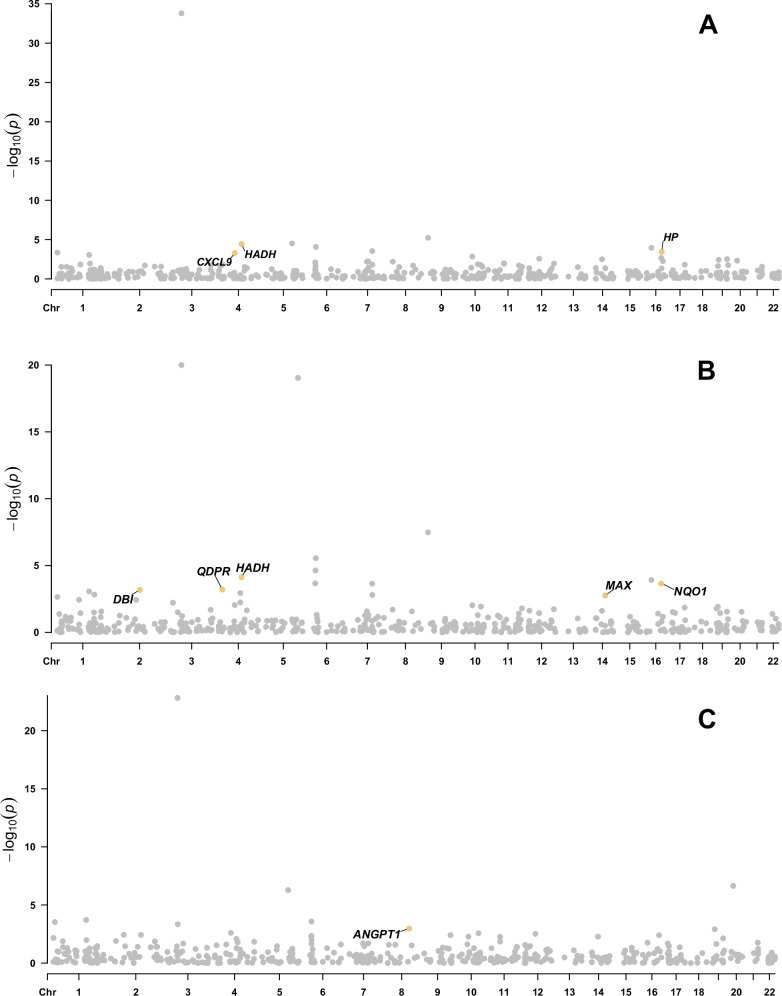
Manhattan plot for associations between genetically predicted mental disorder-related gene encoded protein abundance and risk of IBD A, UC B and CD C. Genes passing multiple corrections (*P* < 0.05) and colocalization threshold (PPH4 > 0.7) were labled. IBD inflammatory bowel disease, CD Crohn’s disease, UC ulcerative colitis.

**Fig. 4:**
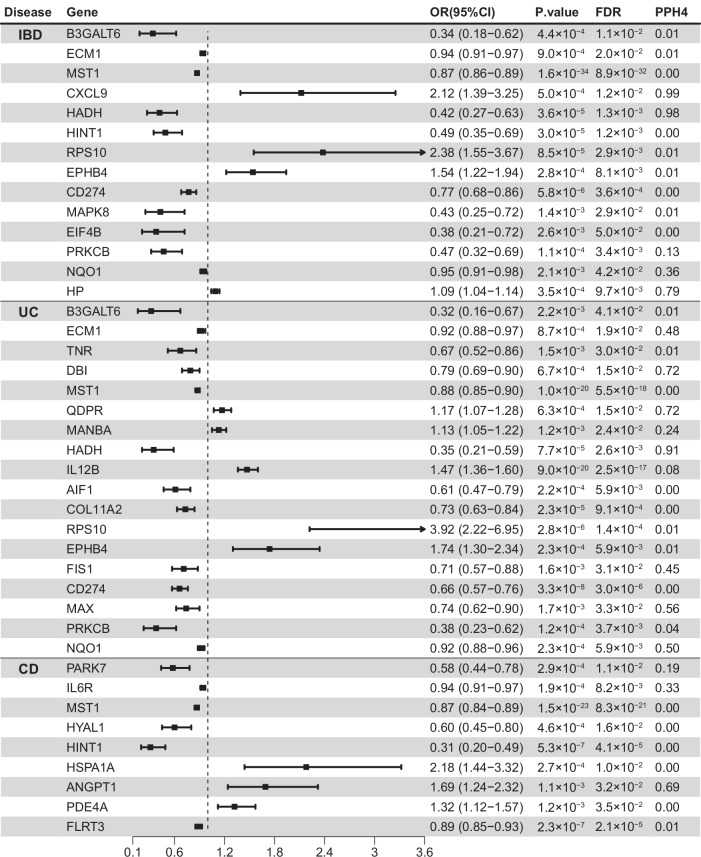
Associations between genetically predicted mental disorder-related gene encoded protein abundance and risk of IBD and its subtypes in summary-data-based Mendelian randomization analysis. IBD inflammatory bowel disease, OR odds ratio, CI confidence interval, PPH4 posterior probability of H4.

At an FDR-corrected *P* < 0.05 level, 14, 18, 9 mental disorder-related gene encoded proteins were significantly associated with IBD, UC, CD respectively (Table S5). Genetically predicted higher level of *HADH* (OR = 0.42, 95%CI: 0.27–0.63, PPH4 = 0.98) was associated with a decreased risk of IBD, while higher levels of *CXCL9* (OR = 2.12, 95%CI: 1.39–3.25, PPH4 = 0.99) and *HP* (OR = 1.09, 95%CI: 1.04–1.14, PPH4 = 0.79) were associated with an increased risk of IBD. Meanwhile, genetically predicted higher levels of *DBI* (OR = 0.79, 95%CI: 0.69–0.90,PPH4 = 0.72), *HADH* (OR = 0.35, 95%CI: 0.21–0.59, PPH4 = 0.91), *MAX* (OR = 0.74, 95%CI: 0.62–0.90, PPH4 = 0.56) and NQO1 (OR = 0.92, 95%CI: 0.88–0.96,PPH4 = 0.50) were inversely associated with the risk of UC, while a higher level of *QDPR* (OR = 1.17, 95%CI: 1.07–1.28,PPH4 = 0.72) was positively associated with the risk of UC. Similarly, among those genes passing the threshold of multiple testing and colocalization, genetically predicted level of *ANGPT1* (OR = 1.69, 95%CI: 1.24–2.32, PPH4 = 0.69) was positively associated with the risk of CD (Figs. 3 and 4). Associations for *CD274*, *RPS10*, *MST1*, *COL11A2* were replicated in the FinnGen study (Table S6).

### Integrating evidence from multi-omics level

By integrating evidence from multi-omics perspectives, we identified four mental disorder-related genes: *QDPR* (Tier 1), *DBI* (Tier 1)*, MAX* (Tier 3) and *HP* (Tier 3) as prior regulatory genes in the pathogenic pathway of IBD and its subtypes (Table 1). Consistency in directionality of effect estimates was observed across both eQTL-GWAS and pQTL-GWAS analyses for all tier genes. Similarly, coherence in directionality of effect estimates was maintained for distinct CpG sites within the same gene. There were inverse associations between gene methylation and expression in *QDPR* (cg08808571, cg26689483), consistent with their deleterious role in UC. Conversely, the methylation of *DBI* (cg11066750) exhibited a protective effect on UC through the enhancement of gene expression (Table S7). Identified associations based on UK Biobank and FinnGen studies remained consistent with results of the discovery stage in effect direction except for *HP* gene (Table S8).

**Table 1:** Tier of genetically predicted methylation, expression, and protein of candidate gene with IBD and its subtype in summary-data-based Mendelian randomization analysis.

Outcome	Gene	Tier	mQTL			eQTL		pQTL	
			Probe	OR (95%CI)	*P*-value	OR (95%CI)	*P*-value	OR (95%CI)	*P*-value
UC	QDPR	Tier 1	cg08808571	0.93 (0.89–0.97)	1.64E-03*	1.07 (1.03–1.11)	1.19E-03*	1.17 (1.07–1.28)	6.27E-04*
			cg26689483	0.95 (0.92–0.98)	1.55E-03*				
	DBI	Tier 1	cg11066750	0.88 (0.82–0.95)	1.41E-03*	0.84 (0.75–0.93)	9.34E-04*	0.79 (0.69–0.90)	6.70E-04*
	MAX	Tier 3	cg16583315	0.93 (0.89–0.97)	1.54E-03*	0.81 (0.69–0.95)	9.53E-03	0.74 (0.62–0.90)	1.72E-03*
IBD	HP	Tier 3	cg04162276	0.85 (0.75–0.96)	6.77E-03	1.05 (1.01–1.09)	1.18E-02	1.09 (1.04–1.14)	3.52E-04**
			cg23815491	0.97 (0.95–0.99)	1.19E-02				

*QTL* quantitative trait loci, *CI* confidence interval, *OR* odds ratio, *FDR* false discovery rate, *IBD* inflammatory bowel disease, *UC* ulcerative colitis, *CD* crohn’s disease.

*FDR < 0.05, **FDR < 0.01.

### Intestine tissue validation

Genetically predicted gene expression of *QDPR* showed a positive effect across transverse colon (OR = 1.14, 95%CI: 1.05–1.24), small intestinal terminal ileum (OR = 1.21, 95%CI: 1.06–1.38) and meta intestine tissue (OR = 1.24, 95%CI: 1.08–1.43) (Table S9). The direction of associations validated in the discovery phase remained consistent with the results. However, we were unable to validate the associations of *DBI*, *MAX and HP* gene expression with the diseases due to a lack of corresponding instrumental variables in certain tissues.

## Discussion

In this study, we employed SMR and colocalization approaches to elucidate key genes playing crucial roles in the gut-brain interaction pathway for IBD, starting from the genetic prediction of gene methylation, expression, and protein abundance. We identified four key genes and their downstream pathways, specifically *QDPR*, *DBI*, *MAX* and *HP* genes, linking the mental disorders and susceptibility to the development of IBD.

*QDPR*, also known as *DHPR*, encoded dihydropteridine reductase, an enzyme that catalyzes the NADH-mediated tetrahydrobiopterin (BH4) regeneration from dihydrobiopterin (BH2), involving the reduction of nicotinamide adenine dinucleotide (NADH) [34]. In addition, as a cofactor, BH4 also played a crucial part in the synthesis of key neurotransmitters like dopamine, serotonin and nitric oxide synthases [34]. Evidence indicated that BH4 is closely related to a variety of neuropsychiatric disorders such as Alzheimer’s disease, ASD and depression [35–37]. The relationship between *QDPR* and UC remained ambiguous. A recent experiment suggested that BH4 attenuated DSS-induced colitis in mice by balancing REDOX and lipid signaling pathways [38]. Although the demand for BH4 in the gut was likely to be met mainly by de novo synthesis at a rate controlled by guanosine triphosphate cyclohydrolase rather than *QDPR* [38], our study highlighted the importance of *QDPR* in the development of UC.

In our study, *DBI* demonstrated a protective effect against UC at both the gene expression and protein levels. *DBI* encoded Acyl-coenzyme A binding protein (ACBP), also known as diazepam binding inhibitor, which was identified to displace diazepam from its binding site on GABA_A_ receptor and modulate its activity [39]. Current researches indicated that *DBI* was associated with some mental disorders like anxiety and autism spectrum disorder [40–42]. A recent animal experiment had demonstrated that neutralizing of *ACBP/DBI* resulted in an “antidepressant” effect [43]. Although intracerebroventricular administration of neuropeptides derived from *ACBP/DBI* can induce anxiety in rodents [44], a study had shown that endogenous ACBP did not affect anxiety-like behaviors, and *ACBP* knockout impaired the anxiolytic responses to diazepam in mice [45]. In addition, mice with *ACBP/DBI* knockout exhibited a lack of social interest and repetitive self-grooming behavior, which was part of autism-like features [46]. Direct evidence on the association between *DBI* and UC was rare. A study demonstrated *DBI* gene were scarce in the human gut microbiome [47]. Previous studies had elucidated the pivotal involvement of autophagy in the pathogenesis of UC [48, 49]. There was compelling evidence indicating an interrelation between *DBI* and autophagy [50], postulating a possible mechanistic linkage between *DBI* and the pathogenesis of UC. In the future, more observational and experimental studies were imperative to support and extend our findings.

The findings in our study suggested that the low methylations of cg04162276 and cg23815491 and high gene expression in *HP* were associated with an increased risk of IBD at a nominal significance. And *HP* showed a statistically positive association with IBD risk at protein levels. *HP* gene, harboring a polymorphism of two common alleles: *HP1* and *HP2*, encoded haptoglobin and its related protein Zonulin, a physiology mediator to regulate intestinal permeability intestinal permeability through inducing tight junction [51–53]. Intestinal permeability was considered as one of key pathogenesis of inflammatory bowel disease [54]. A research showed that experimental colitis were more severe in *HP*-KO mice, suggesting *HP* played an modulatory and protective role in inflammatory colitis by inhibiting the production of several cytokines [55]. In accordance with this author’s reasonable speculation, the *HP* gene in WT mice was similar to the human *HP1* allele, while *HP*-KO was similar to the human *HP2* allele [55]. Therefore, *HP2* demonstrated a risk factor of human IBD. Similarly, both in vivo and in vitro measurements showed increased intestinal permeability at baseline in zonulin transgenic *HP2* mice compared to wild-type mice (*HP1*), which was exacerbated by DSS treatment and associated with an up-regulation of *HP* gene expression [56]. A genotyping of 1 061 patients with CD and 755 patients with UC exhibited prevalence of *HP2* was higher in CD and UC than in controls [55]. Likewise, one study had observed an elevated level of fecal and serum zonulin in patients with active CD [57]. The above-mentioned evidence from animal and population sequencing further corroborated the potential association of *HP* with an increased risk of IBD.

*MAX* encoded Myc Associated Factor X which was a transcriptional regulator and an essential heterodimerization partner for the Myc oncoproteins [58]. It played a critical role in cellular processes such as proliferation, differentiation, and apoptosis [59], and therefore was regarded as one of the key factors in the development of colitis-associated colorectal cancer [60]. Recent studies had highlighted the relevance of *MAX* in psychological disorders [61]. For instance, social defeat induced a depression-like phenotype in male adolescent mice, accompanied by significantly elevated levels of MAX protein in the hippocampus [62]. 10058-F4 is a compound known to inhibit MAX heterodimerization, which, in other words, disrupts the interaction between c-Myc and MAX [63]. A recent study demonstrated that the Myc inhibitor 10058-F4 partially reversed the detrimental effects of Treg cell-specific CD226 deficiency in the DSS-induced colitis model [64]. These findings and our results suggested that MAX may play a protective role in colitis by modulating immune responses and its interaction with Myc, offering instructive insights into its involvement in both psychological disorders and inflammatory diseases like UC.

The most prominent strength of our research lies in the integration of multi-omics evidence from mQTL, eQTL and pQTL, which significantly enhance the reliability of our conclusions and unveil possible biological pathways. Moreover, MR design and colocalization were employed to estimate effects of candidate genes on IBD, aiding in the identification of causal associations and causal variants by diminishing bias from confounding, reverse causality and linkage disequilibrium. Additionally, the concordance of our findings across various datasets served to bolster the robustness of our conclusions. However, some limitations should be acknowledged in our study. Firstly, we opted to analyze only the genes associated with six primary mental disorders, potentially overlooking other crucial genes implicated in the gut-brain axis. Secondly, given the limited numbers of mental disorder-related proteins in the pQTL dataset, some genes could not be validated at the protein level. Additionally, the lack of corresponding instrumental variables in intestinal tissues also hindered the validation of some genes. Thirdly, both the discovery and replication phases in this study were conducted in populations of European ancestry, thereby constraining the generalizability of the findings to other ethnic groups.

## Conclusion

This study employed QTLs as instrumental variables to estimate the causal effects of gene methylation, expression, and protein abundance related to mental disorders on IBD and its clinical subtypes. Our findings provided a multi-omics perspective on the association between mental disorders and IBD. We identified four mental disorder-related genes, i.e., *QDPR*, *DBI*, *MAX* and *HP* and their downstream pathways that played crucial role in the susceptibility of IBD and UC, suggesting their potential as intervention and therapeutic targets for gut-brain axis diseases in the future.

## Supplementary information

Fig S1.

Supplemental tables

## Data Availability

The sources of all summary level data used in this study are comprehensively explained in the Method section. All data used in this study were publicly accessible.

## Code availability

The analysis code used for data analysis is available from the corresponding author on reasonable request.
