# Supplementary material for: Multi-omics insight into the molecular networks of mental disorder related genetic pathways in the pathogenesis of inflammatory bowel disease
Source: Transl Psychiatry. 2025 Mar 21;15:91. doi: 10.1038/s41398-025-03299-2 (PMC11928517; doi:10.1038/s41398-025-03299-2)
Supplement: Supplementary file 1 — Fig S1. [file 41398_2025_3299_MOESM1_ESM.pdf]

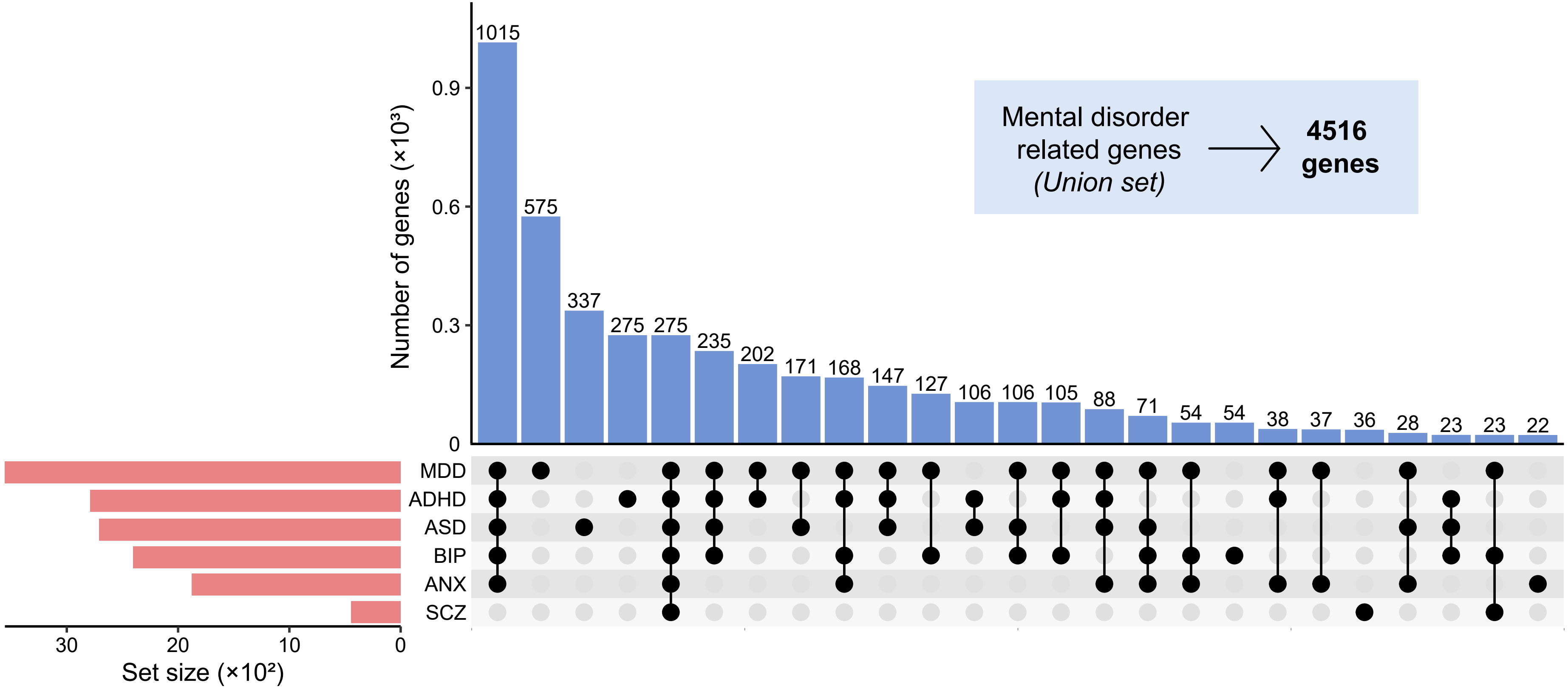

Fig S1. Candidate mental disorder-related genes. The UpSet plot exhibited the intersection of genes of six mental disorders. The union of six sets was identified candidate genes.

Abbreviation: MDD, major depressive disorder, ANX, anxiety disorder, ADHD attention-deficit/hyperactivity disorder, ASD, autism spectrum disorder, BIP, bipolar disorder, SCZ, schizophrenia.
